# Anticipating Clinical Onset in Multiple Sclerosis: Challenges and Opportunities

**DOI:** 10.3390/jpm15120598

**Published:** 2025-12-04

**Authors:** Viviana Nociti, Marina Romozzi

**Affiliations:** 1 Neurologia, Dipartimento di Neuroscienze, Organi di Senso e Torace, Fondazione Policlinico Universitario Agostino Gemelli IRCCS, 00168 Rome, Italy; marina.romozzi@unicatt.it; 2 Centro Sclerosi Multipla, Università Cattolica del Sacro Cuore, 00168 Rome, Italy

**Keywords:** disease-modifying therapies, radiologically isolated syndrome, biomarkers

## Abstract

Multiple sclerosis (MS) is a chronic, immune-mediated disorder of the central nervous system, increasingly recognized as a disease continuum that begins years before the first neurological event. Genetic susceptibility, environmental exposures, and silent neuroinflammation contribute to early disease activity. Recent studies have highlighted a preclinical phase that includes both a biological stage, characterized by elevated biomarkers such as serum neurofilament light chain up to 10 years before onset, and a prodromal phase, marked by subtle but measurable symptoms. Population-based cohorts consistently show increased healthcare use, higher prevalence of psychiatric and cognitive disturbances, fatigue, pain, and gastrointestinal disorders years before diagnosis, which may represent prodromal symptoms. Radiologically isolated syndrome (RIS), defined by incidental demyelinating lesions in asymptomatic individuals, represents the visible form of this phase and provides a unique opportunity to study the transition to clinical disease. Approximately half of RIS patients develop MS within a decade, with predictors including younger age, male sex, CSF oligoclonal bands, and spinal cord involvement. Recent randomized controlled trials demonstrated that early use of disease-modifying therapies in RIS significantly reduces conversion risk. Defining the preclinical and prodromal phases of MS offers a major opportunity to refine risk stratification, enable earlier intervention, and ultimately prevent or delay the onset of clinically definite MS.

## 1. Introduction

Multiple sclerosis (MS) is an inflammatory and degenerative disease of the central nervous system (CNS), characterized by broad intra- and interindividual heterogeneity [1]. So far, the pathophysiology of MS appears to be characterized by an abnormal immune activation that leads to neuroinflammation, in which both peripheral immune cells and resident cells of the CNS (e.g., astrocytes and microglia) are involved [1].

Multiple sclerosis arises from the convergence of genetic predisposition, environmental exposures, and complex biological mechanisms [2].

The development of MS is thought to start in genetically susceptible individuals [3]. Genetic epidemiological studies have shown the importance of genetic factors in susceptibility to MS. Family studies assessing risks to relatives of MS probands have revealed a marked familial aggregation of this disease [3].

The 2024 revisions of the McDonald criteria allow for an early diagnosis of MS and a more accurate diagnosis by integrating novel biomarkers, expanding topographic regions, reducing misdiagnosis risk, and allowing for earlier treatment initiation, thus improving outcomes and global applicability across diverse clinical scenarios [4,5].

Clinically isolated syndrome (CIS) describes a first episode of neurological symptoms consistent with a demyelinating etiology and suggestive of MS [4].

Regarding definite MS, approximately 85% of patients present at onset with a relapsing-remitting MS (RRMS), defined by episodes of acute exacerbations followed by a return to the status quo. Most RRMS patients develop a secondary progressive form of MS (SPMS), characterized by a gradual worsening in neurological functions with or without relapses. In circa 10% of the cases, patients present a primarily progressive course (PPMS), defined by a progressive accrual of disability since the onset of the disease [6].

However, in the past few years, MS has been increasingly recognized not only as a clinical disease entity but also as the culmination of a long and complex biological process that begins years before the onset of neurological symptoms [7].

In this context, another entity, radiologically isolated syndrome (RIS), has gained considerable importance, as it provides a window into the preclinical stage of MS and an opportunity for early interventional strategies [8]. It was first described in 2009 by Okuda et al., and it is defined as the presence of asymptomatic, incidentally identified demyelinating-appearing white matter lesions in the CNS within individuals lacking symptoms typical of MS [9,10]. Approximately 30% of adult patients with RIS convert to clinical definite MS, and 50% at 10 years, with younger age, male sex, presence of CSF oligoclonal bands, and spinal cord involvement appearing to be the most important independent predictors of symptom onset [11,12].

**Figure 1:**
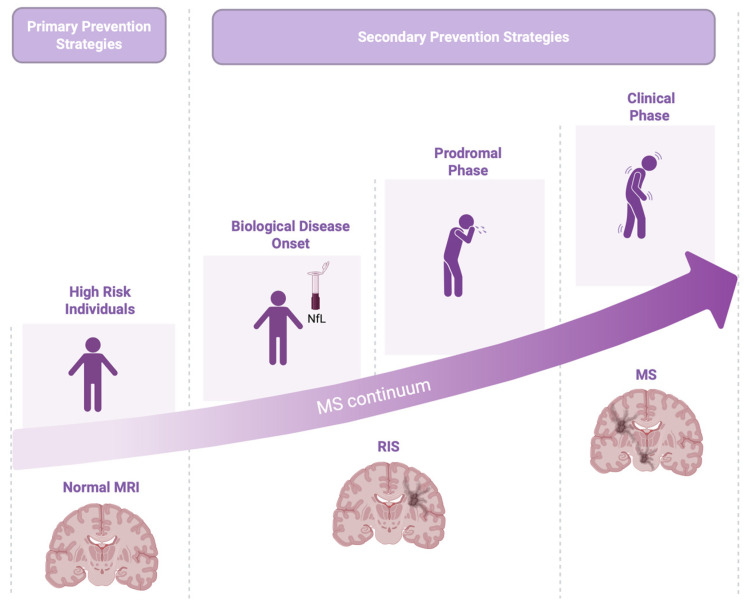
Multiple sclerosis (MS) biological continuum.

Recent work has reframed MS within a continuum spanning from health in at-risk individuals to clinically definite MS. The concept of a biological continuum suggests that MS originates from a complex interaction of genetic and environmental factors, progresses through silent biological activity and prodromal stage, until the clinical onset [7] (Figure 1).

This continuum includes phases such as asymptomatic high-risk states, RIS, and the prodromal phase, ultimately culminating in CIS and definitive MS [7]. Perhaps it includes a “subclinical stage” that is asymptomatic and a clinical phase, passing through a prodromal stage where the first nonspecific symptoms appear [13].

Framing MS in this way is essential, as it opens the door to earlier recognition, biomarker development, monitoring, and potentially preventive interventions, potentially moving beyond the traditional model of waiting for the first neurological attack to initiate DMTs [7].

This article aimed to review the current knowledge on the preclinical phase of MS.

## 2. Materials and Methods

To conduct this narrative review, in order to capture all the relevant articles on the topic, PubMed was searched using the terms ‘multiple sclerosis’ and ‘prodrome’, ‘prodromal symptoms’, ‘preclinical phase’, ‘biological onset’, and ‘radiologically isolated syndrome’. The bibliographies of all relevant papers and reviews were hand-searched for additional articles. The data were extracted into spreadsheets.

## 3. High-Risk Population and Risk Factors

The population with a higher risk of developing MS is represented by family members of PwMS [14]. First-degree relatives are generally 10 times more at risk of developing MS than the general population [14]. More specifically, the probability of developing MS is 30% in monozygotic twins, 3% in first-degree relatives and dizygotic twins, and 1% in second-degree relatives [3]. Furthermore, first-degree asymptomatic family members may already have subtle neurological manifestations and radiological evidence of disease [15]. The study by Xia Z. et al. on asymptomatic high-risk relatives of MS patients demonstrated that this population has an increased likelihood of harboring early subclinical manifestations and radiological lesions, highlighting not only the importance of active surveillance for early diagnosis, but also the potential for implementing secondary prevention strategies [15].

Environmental factors also play a pivotal role in modulating disease initiation: Epstein–Barr virus (EBV) infection is strongly associated with MS development, and tobacco smoke exposure further amplifies risk [16]. In addition, low sunlight exposure and inadequate vitamin D levels compromise immune regulation, while obesity during childhood or adolescence exerts a pro-inflammatory effect that predisposes to a disbalance of the immune system [17].

Air pollution, oxidative damage, chronic inflammation, and hormonal changes are more strongly associated with late-onset MS [18].

## 4. The Biological Phase of Multiple Sclerosis

The complex interplay between genetic and environmental risk factors triggers the biological phase of MS, characterized by an immune-mediated attack against the CNS through “silent” inflammation, which initiates demyelination and neurodegeneration in an as yet asymptomatic individual. At this stage, there is already a biological marker indicative of ongoing axonal damage: neurofilament light chain (NfL). Being key components of the neuronal cytoskeleton, neurofilaments are released into the CSF and, to a lesser extent, the blood, following axonal injury. In a nested case–control study conducted on veterans, serum NfL (sNfL) levels were increased up to six years prior to symptom onset in individuals who later developed MS compared with matched controls [19]. Similarly, the study by Jons D. et al. conducted in a larger cohort (519 presymptomatic individuals who later received an MS diagnosis) confirmed that sNfL levels are significantly elevated up to 10 years before clinical onset [20]. The trajectory of sNfL values follows a gradual progression that precedes the appearance of the first symptoms. Thus, there exists a long temporal window in which the disease is biologically active before reaching the “clinical threshold.” The phase following the biological stage is the prodromal phase.

## 5. Multiple Sclerosis Prodromes

**Figure 2:**
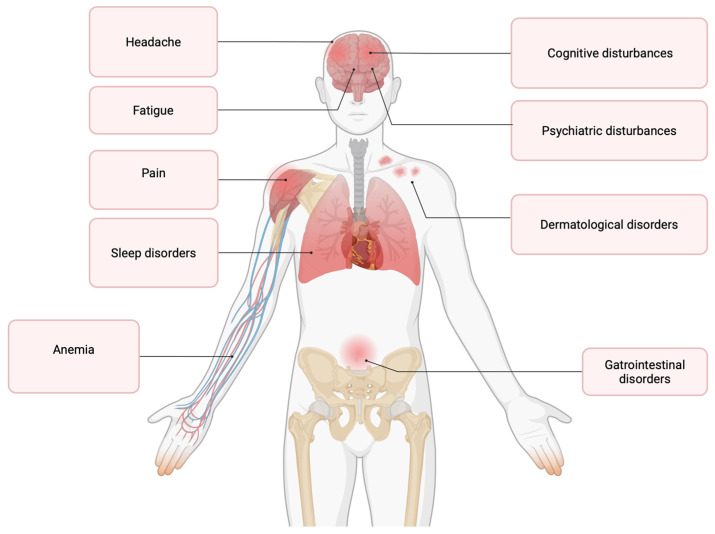
Prodromal multiple sclerosis (MS) symptoms.

A prodromal phase can be defined as an early set of signs or symptoms that indicate the onset of a disease before more typical symptoms develop [21]. Evidence for a prodrome in MS has been accumulating for some years, and it has been suggested from Registries and studies regarding healthcare utilization (Figure 2) [21].

It has been shown that annual healthcare use steadily increased in the five years prior to the diagnosis of MS compared to the general population. In a Canadian study from a health administrative cohort including 14,428 PwMS and 72,059 matched controls, it was found that annual healthcare use increased steadily between 5 years and 1 year before the first demyelinating disease compared with the general population [22].

In particular, in the year before the first demyelinating event, PwMS had a 78% higher rate of hospitalizations, 88% higher rate of physician visits, and a 49% increase in the number of prescribed drug classes compared to controls [22].

Another Canadian study on 4092 PwMS showed that they had 15% more physician visits than matched controls in the 5 years before MS diagnosis [23].

A study conducted in the UK to explore the medical records of 10,204 individuals who were later diagnosed with MS reported a wide range of symptoms, including headache, gastrointestinal, intestinal, urinary, and anorectal problems, pain, and psychiatric conditions up to 10 years before diagnosis, and fatigue up to 5 years before [13].

Similarly, there was a significant increase in sickness absence spells in individuals on the path to developing MS [24].

All these findings point to the existence of a prodromal stage of MS, which refers to a period during which individuals experience subtle, nonspecific symptoms years before the first demyelinating event.

This may suggest that subclinical immune dysregulation and microglial activation may precede radiological conversion to MS, contributing to early systemic manifestations such as fatigue, cognitive, and mood disturbances. The interplay between peripheral immune activation, microglial priming, and synaptic dysfunction may drive subtle network alterations before overt demyelination becomes detectable [25].

### 5.1. Cognitive Disturbances

Studies on cognitive performance before the onset of MS symptoms have reported inconsistent results [26]. In a study from the Norwegian MS Registry, in 924 PwMS, cognitive performance was lower than that of the 19,530 healthy controls up to 2 years before MS onset. Among the small percentage of individuals who developed PPMS, cognitive performance was lower than that of the controls up to 20 years before the clinical onset of MS. However, these cohorts included only men [26].

A separate study found that high school students who eventually developed MS had significantly poorer academic performance in their final school years compared to peers who did not develop the disease. Notably, educational outcomes in the last year of school were associated with the interval to MS clinical onset, indicating possible prodromal disease activity in those nearing symptom manifestation [27].

Two small studies found a similar cognitive profile of patients with RIS and MS [28,29].

In particular, in the study from the Italian RIS/MS study group on 29 patients with RIS and 25 with MS, the authors found a similar profile of cognitive deficits observed in patients with RRMS in 27% subjects with RIS [29].

On the other hand, a Swedish nested case–control study including men from the Military Conscription Register did not find a significant difference in cognitive profile between patients and controls [30].

Cognitive dysfunction may precede the first clear relapse. Diffuse pathological processes in the brain might increasingly disrupt neuronal connectivity and lead to subtle cognitive problems that are compensated for in daily life but may be unmasked by detailed neuropsychological testing [29].

### 5.2. Psychiatric Disturbances

Psychiatric manifestations, particularly mood and anxiety disorders, frequently occur in the years preceding the clinical onset of multiple sclerosis (MS) and may represent features of its prodromal phase [31]. This association was first suggested nearly three decades ago, when a study involving 45 patients with MS reported that 52% had experienced at least one depressive episode before disease onset [32]. More recently, population-based data from Canada confirmed an excess of psychiatric conditions—such as depression, anxiety, schizophrenia, and bipolar disorder—among individuals with MS. Specifically, psychiatric morbidity was 91% higher in 6863 MS cases during the five years prior to their first demyelinating event compared with 31,865 matched controls, and 58% higher in 966 MS cases during the five years before symptom onset relative to 4534 controls [31]. A subsequent analysis of the same cohort showed that mood and anxiety disorders were significantly more prevalent in MS cases than in controls during the five years preceding either a first demyelinating event (24% vs. 15%) or the appearance of initial MS symptoms (19% vs. 15%) [33].

Consistent findings were also reported by Hoang and colleagues, who observed an increased risk of anxiety and depression in the two years prior to MS diagnosis, though no significant elevation was noted in the two years following diagnosis. This psychiatric vulnerability was further reflected in higher prescription rates for psychotropic medications, including tricyclic antidepressants and selective serotonin reuptake inhibitors (SSRIs) [34].

### 5.3. Headache

Headache was described as a potential MS prodrome. A few studies, mostly retrospective on small sample sizes, have reported that migraine precedes MS onset or diagnosis by 7–8 years on average [35,36].

In a recent study, among the 591 PwMS, 21.2% developed migraine concurrent with or before the onset of MS compared to 18.7% of the 651 controls. Migraine had to fulfil the International Classification of Headache Disorders, 3rd edition (ICHD-3) criteria. Migraine onset was more likely to occur either concurrently with MS, and the authors hypothesized that headache with migraine-like features could be part of the constellation of MS onset symptoms rather than a risk factor for or prodromal symptom of MS [37].

A study from the Nurses’ Health Study found that the relative risk of developing MS in patients with migraine was 1.39 times higher than in people without a history of migraine [38]. On the other hand, the risk of reporting non-migraine headaches seems not to be increased in women before MS onset compared to women who did not develop MS [39].

However, considering the high prevalence and variations in different latitudes of migraine, it is difficult to establish a conclusion on this association. However, neuroinflammation is a key component of MS pathophysiology and also plays an important role in migraine. Early neuroinflammatory changes may already occur during the prodromal phase, potentially giving rise to headache or migraine in a preclinical stage [40]. Longitudinal studies carefully adjusted for confounders are needed to clarify this relationship.

### 5.4. Other Symptoms

A large population-based analysis identified fatigue, sleep disturbances, anemia, and pain as elements of the MS prodrome. Among these, pain emerged as the most frequent symptom during the five years preceding either the first demyelinating event or the initial manifestation of MS, followed in prevalence by sleep disorders, anemia, and fatigue. Notably, the risk of sleep disorders was elevated by 72–161% in individuals with MS compared to controls, while the likelihood of pain was 53–115% higher [41].

In Berger et al., it was reported that fatigue was diagnosed before MS onset in 28.9% of patients (*n* = 1534). Of these, 10.4% received pharmacological treatment specifically for fatigue. In nearly 40% of patients, fatigue represented the earliest clinical symptom, and 30.8% experienced fatigue as their sole complaint during the three years prior to their first MS diagnosis [42].

Further evidence from a Canadian study revealed increased dermatology consultations during the prodromal period, particularly among individuals who later developed relapsing–remitting MS (RRMS, *n* = 1887) compared with those who progressed to primary progressive MS (PPMS, *n* = 171) in the five years before initial symptoms. These dermatological manifestations may reflect early systemic inflammation associated with MS [43].

Gastrointestinal manifestations have also been linked to the prodromal phase. A UK nested case–control study demonstrated a higher prevalence of gastrointestinal complaints in MS cases than in matched controls up to a decade before the first diagnostic code for MS or CIS [13].

Similarly, a recent Canadian study reported that individuals who later developed MS sought medical care more frequently for gastritis, duodenitis, and esophageal disorders during the pre-diagnostic years [44]. These observations are noteworthy in light of hypotheses implicating gut–brain axis dysregulation in MS pathogenesis [44].

Reproductive health also appears to be altered in the prodromal period. In the five years prior to symptom onset, women with MS had fewer pregnancies and a higher use of hormonal contraceptives compared to matched controls, with these differences being most pronounced in the year immediately before onset. Such findings may reflect behavioral changes, whereby women postpone pregnancy in response to unexplained early symptoms [22].

## 6. Radiologically Isolated Syndrome

The “visible form” of the biological and prodromal phase is represented by RIS. The recognition of RIS as the preclinical phase of MS allows anticipation of the clinical diagnosis of the disease; this represents a major opportunity to implement secondary prevention strategies and avoid progression to overt MS [45]. However, the classification of RIS subjects according to their risk of conversion, as well as their therapeutic management, remains a matter of considerable debate [45].

Incidental findings of demyelinating lesions suggestive of MS in individuals with no clinical symptoms or signs of disease during their lifetime are well documented in several post-mortem studies. Neuropathological investigations have shown that demyelinating lesions may remain clinically silent throughout life in a significant proportion of individuals (approximately 0.1–0.3% in autopsy studies) [10].

The absence of clinical manifestations may be explained by lesion localization in clinically silent CNS areas, a low degree of inflammation, or the individual’s functional reserve [46]. Moreover, the widespread use of MRI as a standard tool for in vivo CNS assessment has further increased incidental detection of alterations possibly of demyelinating origin, though with uncertain clinical significance. A meta-analysis of 16 studies involving 15,000 subjects reported incidental findings of inflammatory CNS lesions in <0.1% of cases [47]. This rate increases to 4% among asymptomatic first-degree relatives of patients with sporadic MS, and up to 10% among those with familial MS [48].

In 2009, Okuda et al. [10] formally defined this new entity as “radiologically isolated syndrome,” and in 2023, Lebrun-Frénay et al. [9] revised its diagnostic criteria. RIS is a rare condition, affecting approximately 0.05% of the general population [46].

The main novelty of the revised framework for RIS lies in its formal integration into the 2024 McDonald diagnostic criteria, representing a major conceptual evolution from previous definitions. Historically, RIS was considered an at-risk but asymptomatic radiological finding. In contrast, the new recommendations acknowledge RIS as part of the MS disease continuum. For the first time, RIS can meet criteria for MS diagnosis when specific combinations of biomarkers are present—namely, DIS with either DIT, positive CSF (OCB or kFLC), or central vein sign.

In a cohort of 451 RIS patients (86% female; mean age at diagnosis 37.2 years), the estimated risk of developing a first clinical event within 5 years was 34%. Independent predictors were male sex, younger age, and spinal cord lesions at baseline MRI [12]. Fifteen patients in the same cohort developed progressive MS within a median of 3.5 years; risk factors for progression included male sex, older age, and higher spinal cord lesion load [49]. The cumulative probability of a first clinical event at 10 years was 51.2% in this cohort. Independent predictors of conversion included age, presence of CSF oligoclonal bands (OCBs), infratentorial and spinal cord lesions, and gadolinium-enhancing lesions at follow-up. Furthermore, Rival et al. demonstrated that elevated serum neurofilament light chain (sNfL) (sNfL > 5 pg/mL) or CSF NfL (>260 pg/mL) levels at RIS diagnosis were strongly associated with disease activity and clinical conversion [50]. In addition, a study of 61 children with RIS followed longitudinally for a mean of 4.2 ± 4.7 years further reinforced the prognostic value of OCBs, whose presence increased the specificity of MRI criteria in predicting subsequent conversion to MS [51].

In a study of 75 individuals with RIS, both unique CSF OCBs and elevated CSF NfL levels were independent predictors of clinical conversion [52].

Beyond sNfL and OCBs, other emerging candidates for biomarkers of conversion to MS include CSF single-cell RNA sequencing, circulating or CSF-derived microRNAs, glial fibrillary acidic protein, and cytokines [53]. In addition, structural biomarkers, such as abnormal visual evoked potentials and retinal thinning on optical coherence tomography, may provide complementary information on the risk of conversion of RIS [54,55].

Taken together, these findings suggest that combining nonspecific symptoms with biological and imaging markers may help define a true prodromal state of MS.

## 7. Implications for Early Diagnosis: Possible Primary and Secondary Prevention

### 7.1. Possible Primary Prevention: High-Risk Population

Family members of PwMS represent the population with a higher risk of developing MS [15]. The recognition of these individuals and the active surveillance may provide an opportunity to prevent the biological phase of the disease through primary prevention.

Primary prevention focuses on modifiable risk factors and includes prevention of Epstein–Barr virus (EBV) infection (through the development of a specific vaccine), increased sun exposure, vitamin D supplementation, smoking cessation, and regular physical activity [16].

### 7.2. Possible Secondary Prevention: Prodromal Phase and RIS

The recognition of a prodromal phase in MS may provide an opportunity for earlier diagnosis and intervention [56]. However, identifying this phase clinically is extremely challenging. Symptoms that frequently occur before disease onset, such as migraine, fatigue, or psychiatric disturbances, are nonspecific and very common in the general population. Even if these symptoms are nonspecific, when present in individuals at risk, for example, those with a family history, they may indicate the onset of an active disease and warrant further investigations and monitoring [57]. Instead, in the general population, clinical manifestations alone cannot reliably define a prodrome, and the search for biological and imaging biomarkers is crucial to improve specificity and accuracy [56].

Radiologically isolated syndrome represents a unique clinical scenario to investigate the prodromal/preclinical phase of MS. In this scenario, biomarkers are even more crucial for determining which patients may be at risk of converting to definite MS, thereby stratifying patients according to the risk of conversion [45].

In 2023, the results of two clinical trials in RIS populations were published [58,59]. In the first trial (NCT02739542), dimethyl fumarate versus placebo reduced the risk of conversion from RIS to MS by 80% in the treated group compared with controls [58]. In the second trial (NCT03122652), teriflunomide similarly reduced the risk of conversion by 72% compared with placebo [59]. A third trial (NCT04877457) is currently ongoing, testing early high-efficacy treatment with ocrelizumab in RIS, and results are awaited.

These findings, together with the new 2024 diagnostic criteria that allow an MS diagnosis in RIS when certain supportive features are present, highlight the importance of RIS as a model for studying the MS prodrome and as a potential target for early secondary preventive strategies.

Secondary prevention, in addition to managing modifiable risk factors, relies on early disease detection—particularly in the preclinical phase—with the goal of initiating treatment as early as possible to avoid progression to overt MS.

## 8. Conclusions

In conclusion, the identification of individuals at high risk of developing MS—such as asymptomatic relatives of affected patients or those with RIS—offers the opportunity to anticipate the clinical diagnosis and to implement both primary and secondary prevention strategies. These include active surveillance, mitigation of modifiable risk factors, and early treatment with disease-modifying therapies, with the aim of delaying or preventing disease onset.

A critical challenge remains the recognition of the prodromal phase, which is largely defined by nonspecific symptoms common in the general population. For this reason, considerable caution is warranted, and further studies are needed to refine its characterization. Integrating clinical features with imaging and non-imaging biomarkers will be essential to establish a reliable and accurate definition of the MS prodrome.

Such refinement could allow for the stratification of individuals at the highest risk, enabling their enrollment in ethically appropriate clinical trials testing neuroprotective interventions. The ultimate goal is to prevent the onset of clinical MS or to limit long-term disability progression. At the same time, misdiagnosis and overdiagnosis must be carefully avoided, given the nonspecific nature of many early symptoms.

Study heterogeneity, short follow-up durations, and small sample sizes currently limit the generalizability of available findings, highlighting the need for larger and more homogeneous studies.

Overall, a more precise definition of the preclinical and prodromal phases of MS represents a major opportunity: it would improve our understanding of the pathogenic process, identify a therapeutic window for early intervention, and ultimately transform the diagnostic and preventive paradigm of MS.

## Data Availability

No new data were created or analyzed in this study. Data sharing is not applicable to this article.
